# Psychology Doctoral Program Experiences and Student Well-Being, Mental Health, and Optimism During the COVID-19 Pandemic

**DOI:** 10.3389/fpsyg.2021.629205

**Published:** 2021-09-10

**Authors:** Stylianos Syropoulos, Deborah J. Wu, Brooke Burrows, Evelyn Mercado

**Affiliations:** Department of Psychological and Brain Sciences, University of Massachusetts Amherst, Amherst, MA, United States

**Keywords:** well-being, mental health, optimism, graduate students, COVID-19

## Abstract

In a sample of 916 doctoral students from 144 universities across the United States, we examined psychology graduate students’ experiences in their programs, as well as their mental health, well-being, and optimism during the onset of the COVID-19 pandemic. In a path model, we found that students’ psychological experiences in their programs (i.e., social belonging, threat, and challenge) were associated with better mental health and well-being, which in turn was associated with greater optimism about the future during the COVID-19 pandemic. These findings were also corroborated in students’ open-ended responses regarding how COVID-19 has impacted their lives. Findings varied by racial, gender, and sexual identities, as racial minorities, LGBTQ+ students, and women expressed more negative psychological experiences in their programs. We outline suggestions for graduate programs to support their graduate students, which include facilitating social connection, providing encouragement, and emphasizing students’ well-being over their productivity as the current pandemic persists.

## Introduction

Graduate students tend to feel overworked, underpaid, and stressed (Hugo, 2017), and also report lower well-being and poorer mental health than the general population (e.g., Willyard, 2012; Evans et al., 2018). These disparities may be exacerbated during the coronavirus (COVID-19) pandemic due to increases in financial stress and lower access to resources (Zahneis, 2020). Social distancing guidelines and the use of remote learning could also increase feelings of social isolation and loneliness (Killgore et al., 2020; Luchetti et al., 2020), as graduate students continue their studies. Due to these additional stressors, it is important to understand how graduate students can cope and remain resilient during this time. Since prior research has shown that optimism is beneficial in times of difficulty and uncertainty (e.g., Fontaine et al., 1993; Auerbach et al., 2005; Yang et al., 2020) and since graduate students spend a significant amount of their time dedicated to their studies (Lauchlan, 2019), understanding how graduate programs can help students maintain their optimism during tumultuous times such as the COVID-19 pandemic is of crucial importance.

To better understand the impact of graduate programs on their students in the midst of the pandemic, we investigated how graduate students’ psychological experiences in their program (i.e., social belonging, threat, and challenge) impacted their mental health, well-being, and optimism about the future during the COVID-19 pandemic. We placed a particular focus on optimism, as it may be a crucial source of resilience for students while they navigate novel challenges during the pandemic. Furthermore, we investigated the impact of social identities (e.g., race, gender, and sexual orientation) on these variables, as students from historically marginalized backgrounds may face additional challenges in graduate school and during the pandemic. For example, research has shown that BIPOC (Black, Indigenous, and People of Color) students in particular report lower belonging in higher education (e.g., Miller and Orsillo, 2020) and are more likely to experience a disproportionate burden during the COVID-19 pandemic due to existing racial inequities (Croucher et al., 2020; Oppel et al., 2020). In sum, we aimed to investigate the psychological state of graduate students during the pandemic, in recognition of pre-existing stressors inherent to the graduate school experience as well as the additional stressors that those from traditionally marginalized social groups may face. Our intention was to highlight the important role that graduate programs can play in maintaining both the mental health and the academic success of students during troubling times.

## Psychological Experiences in Academics

The type of experiences a student faces in their academic environment exert a great influence on their performance, mental health, and well-being (e.g., Walton and Cohen, 2007; Alter et al., 2010; Cook et al., 2012; Dennehy and Dasgupta, 2017). In the current study, we tested whether individual differences in academic experiences graduate students have in psychology programs impact their mental health, well-being, and optimism during the COVID-19 pandemic. Additionally, we expanded upon previous research by testing whether students from traditionally underrepresented backgrounds (in terms of gender, sexual orientation, race, and ethnicity) reported different levels of these academic experiences.

### Social Belonging

Humans need social connections to thrive (Baumeister and Leary, 1995). In academic environments, members of socially stigmatized minority groups may feel uncertain of whether they truly belong, also known as belonging uncertainty (Walton and Cohen, 2007, 2011). Feeling uncertainty in one’s belonging has been shown to lead to negative academic outcomes. For example, adolescents and college students who feel that they do not belong in their academic settings may express greater feelings of self-doubt, be less motivated, and perform worse in school (Walton and Cohen, 2007, 2011; Cook et al., 2012; Gillen-O’Neel and Fuligni, 2013). In a sample of graduate students from underrepresented racial and ethnic backgrounds, those who reported lower belonging at their university had greater anxiety, stress, and more depressive symptoms (Miller and Orsillo, 2020).

Conversely, previous research has also found that having higher social belonging and support from fellow graduate students predicted lower stress (Clark et al., 2008; Myers et al., 2012) and greater life satisfaction (Tompkins et al., 2016). This is in line with research on how feeling like one belongs to a group can increase perceived social support during times of difficulty, as close relationships can help protect individuals from stress (Cohen, 2004; Uchino, 2009). Graduate school and the COVID-19 pandemic are both periods in which there is heightened stress due to the uncertainty and lack of control experienced (Rettie and Daniels, 2020; Richmond et al., 2019). Thus, having social support from others in their graduate programs, which graduate programs could facilitate, may help protect students’ current mental health, well-being, and optimism during difficult times.

### Threat and Challenge

Threat and challenge are conceptualized as active motivational states and as opposing appraisals in stressful situations (Mendes et al., 2002, 2007). In other words, when in a state of challenge, people feel that they have the resources to be able to overcome the stressor, while those in a state of threat feel unable to do so (White, 2008). Within the academic context, challenge may be conceptualized as the motivation to persist or succeed while threat is the feeling of anxiety or worry about one’s capacity to succeed (Folkman and Lazarus, 1985; Mak et al., 2004; Alter et al., 2010; Dennehy and Dasgupta, 2017). Similar to belonging, the impact of threat and challenge in academic settings have often focused on experiences of stigmatized group members. Members of a negatively stereotyped group may feel stereotype threat, or feel anxiety about possibly confirming the negative stereotype regarding their group, which can lead to decreased academic performance (Steele and Aronson, 1995(for reviews see Schmader et al., 2008; Aronson and McGlone, 2009). However, when students from underrepresented racial and ethnic backgrounds are able to reappraise the threat as a challenge that they can conquer, their academic performance improves (Alter et al., 2010; Jamieson et al., 2010).

Threat and challenge appraisals in academics are also related to mental health and well-being, as greater challenge and lower threat have been associated with lower depressive symptoms (Mak et al., 2004). Outside of the academic context, lower threat and greater challenge have predicted decreased distress, greater psychological well-being, and better health outcomes (Florian et al., 1995; Blascovich, 2008). During the COVID-19 pandemic, psychology graduate students are currently encountering a variety of unprecedented stressors within their programs; classes and meetings going remote, in-person lab research being halted, and limited funding sources. These stressors may be pushing graduate students to learn how to restructure their work, which could further increase their stress and negatively impact their mental health.

## Graduate Student Stress and Mental Health

In a recent report from *Nature*, an international sample of doctoral students across all fields revealed that the majority of graduate students struggle with work/life balance, job uncertainty, concerns with finishing their research in a timely manner, and financial worries (Lauchlan, 2019). Previous research indicated that psychology graduate students also share these similar stressors. A survey conducted by the American Psychological Association (APA) found that 70% of psychology graduate students in the United States, had a major stressor that prevented them from functioning optimally (El-Ghoroury et al., 2012). A majority of graduate students in this sample reported academic pressure, financial trouble, anxiety, and poor work–life balance as key stressors (El-Ghoroury et al., 2012).

Due to these stressors, graduate students may experience declines in mental health. Graduate students who indicated that they struggled with work–life balance also reported more anxiety and depressive symptoms (Evans et al., 2018). Furthermore, graduate students experienced depression and anxiety six times more frequently than the general population, as approximately 40% of graduate students reported moderate to severe depression and anxiety, compared to 18% in the general world population [Anxiety & Depression Association of America (ADAA), 2021]. Rates among psychology graduate programs may be even worse in comparison to other areas of study, as students in the behavioral and social sciences reported greater depression rates in comparison to students in engineering (Allen et al., 2020). Furthermore, an APA survey found that 87% of psychology graduate students reported having anxiety, 68% experienced depressive symptoms, and 19% reported suicidal ideation, all three of which are higher than the national average (Willyard, 2012). However, although these effects have been recorded in previous studies, there is little evidence focusing on graduate student well-being during times of national crisis and times of high uncertainty, such as the current COVID-19 pandemic.

In the current study, we examined psychology graduate students’ mental health and well-being during the current COVID-19 pandemic, a time marked by increased stress and uncertainty (Kluger, 2020; Myers et al., 2020). We sought to investigate how graduate students’ psychological experiences and feelings regarding their program may relate to mental health and well-being, and the tendency to have a more optimistic outlook towards the future during the pandemic.

## Optimism: A Source of Resilience During Times of Uncertainty

Optimism, defined as a measure of positive “generalized outcome expectancies” (Scheier and Carver, 1985) has been considered a hallmark of living a happy life. Studies have found consistent evidence highlighting the positive impact of optimism in different aspects of one’s life. There is ample evidence that optimism is correlated with better health outcomes (e.g., Alarcon et al., 2013; for a review see Scheier and Carver, 2018) and well-being (e.g., Scheier and Carver, 1993). Individuals who tend to be optimistic also perceive negative circumstances as challenges as opposed to threats (Baumgartner et al., 2018), and also exhibit more constructive responses to physical stress (Segerstrom et al., 1998), emotional distress (Kelberer et al., 2018), and work-related stress (Gottschling et al., 2016).

Optimism has been regarded as an effective tool that can help individuals cope with uncertainty. For example, research has shown that when individuals are presented with uncertain events, optimists engage in positive thinking, which in turn helps them interpret the event as more beneficial or advantageous in line with their optimistic worldview (Grover et al., 2019). In times of difficulty, optimism has also been linked to positive outcomes in behavior and cognitive appraisal (e.g., Boldor et al., 2012). For example, optimism during a crisis (e.g., terrorist attack, natural disasters, cancer) is associated with greater resiliency, better mental health, lower stress, and greater engagement in problem-solving (Auerbach et al., 2005; Bleich et al., 2006; Peled et al., 2008; Carbone and Echols, 2017). Even more broadly, optimism has been linked to more adaptive coping (Fontaine et al., 1993; Iwanaga et al., 2004), and greater post-traumatic growth (Britton et al., 2019).

Since optimism is a source of resilience, it is crucial when individuals are faced with uncertainty. Therefore, it is important to understand what gives rise to optimism during difficult situations. In the context of our study, we sought to understand the antecedents of optimism in graduate students during the COVID-19 pandemic in order to identify how graduate programs can help support graduate students. Thus, our current study focuses on how program experiences relate to mental health outcomes and optimism. Since graduate students already struggle with work/life balance, financial worries, and job uncertainty (Lauchlan, 2019), which may be exacerbated during the COVID-19 pandemic, it is important to understand how graduate programs can effectively support high levels of optimism for graduate students, to help students remain resilient during the COVID-19 pandemic.

## The Current Study

In line with recent calls for psychologists to consider the outcomes that the COVID-19 pandemic might have on students (Chenneville and Schwartz-Mette, 2020), the current study examined how graduate students’ psychological experiences in their graduate programs (i.e., belonging, threat, and challenge) correlate with their mental health outcomes and well-being. Importantly, the current study examined how graduate experiences may help foster resilience (i.e., the degree to which they are optimistic about the future) during the onset of the pandemic, a period marked with high uncertainty. Consistent with previous literature (e.g., Mak et al., 2004; Miller and Orsillo, 2020), we hypothesized that positive psychological experiences within a graduate program, defined as higher levels of challenge (i.e., motivation to succeed), and belongingness, and lower levels of threat, would be associated with better mental health and well-being (i.e., greater life satisfaction and subjective happiness, and lower stress and depression). We also hypothesized that students’ who reported better overall mental health and well-being would have greater optimism regarding the onset of the COVID-19 pandemic. Moreover, we tested how students’ social identities of gender, race, and sexual orientation may impact their graduate program experiences, as students with marginalized identities have previously reported more negative experiences in higher education (e.g., Walton and Cohen, 2007; Woodford et al., 2014; Dennehy and Dasgupta, 2017). Lastly, to further contextualize our findings we explored whether students’ open-ended responses to a question focusing on the impact of COVID-19 on their lives, would be associated with their psychological experiences. Based on our findings, we discuss suggestions for how graduate programs can better support their graduate students during this pandemic and future times of crisis, by creating an environment focused on social belonging, positive challenge, and reduced threat which we hypothesize will be associated with greater optimism during the pandemic.

## Materials and Methods

### Participants

Psychology graduate students pursuing doctoral degrees were recruited online (*N* = 1,798). Our recruitment process was initiated on February 14 and lasted until June 4. The sample included in this investigation comprises a sub-sample of students (*N* = 916) who were recruited during the onset of the COVID-19 pandemic from March 23 to April 5, after measures relevant to the pandemic were incorporated into the survey to address the effect of COVID-19 on graduate satisfaction and well-being. Thus, the subsample included here pertains to students who were recruited during the rise of COVID-19 as an ongoing crisis gaining widespread national attention across America.

Graduate students were either contacted directly via a personal email, or indirectly via an email sent to their program’s department chair and/or administrator. They were also able to sign up for the study through a posting in student listservs in different psychological associations.^1^ Our sample consisted of 916 psychology graduate students from 144 different Ph.D. granting institutions in the United States.^2^ Students represented a variety of psychological fields: 321 (35%) were from Clinical, 127 (14%) from Social/Personality, 96 (11%) from Cognitive, 86 (9%) from Developmental, 67 (7%) from Neuroscience, 52 (6%) from Industrial-Organizational, 32 (4%) from Counseling, and 127 (14%) from other fields such as Health and Community Psychology. Students also varied in the number of years they were in graduate school: 155 (22%) were on their first year; 189 (26%) on their second; 13 (2%) on their third, 164 (23%) on their fourth, 131 (18%) on their fifth; and 64 (9%) were in graduate school for more than five years, and 199 preferred to not give a response.

From the 916 students, 772 (85%) were American and 137 (15%) were international. In terms of gender, 701 (77%) students identified as a woman, 193 (21%) as a man and 13 (1%) as transgender or non-binary. For race, 645 (72%) students were White, 107 were Asian or Asian American (12%), 59 (7%) were Latinx, 37 (4%) were African American, and 48 (5%) were multiracial. In terms of sexual orientation, 650 (73%) students were straight, 188 (21%) reported being bisexual/queer, 45 (5%) were gay/lesbian, and 5 (<1%) reported being either asexual or some other sexual orientation. The average age was 27.73 years (SD = 4.16). Students reported low levels of religiosity (*M* = 2.88, SD = 2.37; min = 1, max = 9), were politically liberal (*M* = 2.46, SD = 1.40; min = 1, max = 9) and reported a low annual personal income (*M* = 3.15, SD = 0.81; min = 1, max = 9; equivalent to a range of $10,000--$30,000).^3^

### Materials and Procedure

After providing consent, the students were first presented with a set of demographic questions. Following the completion of the demographic section, participants were presented with the different measures of the study in a randomized order.

### Psychological Experiences in Graduate School

Students’ psychological experiences in graduate school were operationalized and measured as feelings of social belongingness, and perceptions of their graduate program as threatening or challenging. All of the measures included in the study were captured on 1–9 analog slider scales.

#### Social Belonging

Feelings of social belongingness were measured with four items (adapted from Dennehy and Dasgupta, 2017) assessing how much students felt included, accepted, invisible (reverse-coded) or like an outsider within their program (reverse coded), (*a* = 0.90, *M* = 6.30, SD = 1.98).

#### Threat and Challenge

We assessed threat and challenge with a total of eight items (four for each construct, adapted from Dennehy and Dasgupta, 2017). Threat captured the degree to which graduate students felt stressed, worried, unsure about their program, or felt that their program was difficult (*a* = 0.73, *M* = 6.22, SD = 1.49). Challenge captured the degree to which students felt they had the skills and motivations to succeed in graduate school (i.e., “I will be able to overcome any challenges I experience”; *a* = 0.89, *M* = 6.28, SD = 1.53).

### Student Mental Health and Well-Being

#### Mental Health

Mental health was captured by assessing students’ depression and stress levels. Depression (*a* = 0.89, *M* = 4.82, SD = 1.47) was captured with the 10-item version of the Center for Epidemiologic Studies Depression Scale (CES-D Scale; Zhang et al., 2012). Stress (*a* = 0.88, *M* = 5.22, SD = 1.37) was captured with the 10-item Perceived Stress Scale (Cohen and Williamson, 1988).

#### Well-Being

Individual differences in well-being were assessed by measuring students’ satisfaction with life and subjective happiness. How satisfied students felt with their life (*a* = 0.89, *M* = 5.69, SD = 1.66) was measured with the 5-item Satisfaction with Life Scale (Diener et al., 1985). Students’ level of happiness (*a* = 0.91, *M* = 5.66, SD = 1.76) was assessed with the 4-item Subjective Happiness Scale (Lyubomirsky and Lepper, 1999).

### Optimism During COVID-19

Our main outcome focused on students’ optimism for the future. This item was phrased as follows: “*When thinking about the impact of COVID-19 on your life, how optimistic do you feel about the near future?*” Responses ranged from “*1 = Not at all optimistic*” to “*9 = Very optimistic.*” We chose to frame our item to reflect optimism during the COVID-19 pandemic as past research has shown that domain specific measures of optimism are better suited in academic settings in comparison to dispositional optimism measures (Chang et al., 2011). We also chose to utilize a one-item measure of optimism. This choice was made because students were surveyed during the COVID-19 pandemic, a very stressful time, and thus, we wished to keep our survey short to be considerate of students’ time in our recruitment process. Past studies have utilized single-item measures of optimism and have found single item measures to be reliable (Wong and Fielding, 2006; Bentsen et al., 2008; Lau and Knardahl, 2008).

### COVID-19 Open-Ended Question

An additional question, which was open-ended in nature, asked the students how the COVID-19 pandemic had impacted their life.

### Data Analysis Plan

Data management and quantitative analyses were performed in SAS (Statistical Analysis Software) version 9.4. To examine the relationship between students’ psychological experiences in graduate school with their mental health, well-being and optimism for the future, bivariate correlations and path models were computed.

Initial qualitative data analyses were conducted using LIWC (Linguistic Inquiry and Word Count program, see Pennebaker et al., 2015). LIWC calculates the percentage of certain word categories contained within a given response, comparing each word within a text to a specified LIWC dictionary file. For our research, we analyzed participant’s written responses using LIWC’s “positive emotion” and “negative emotion” dictionaries, which contain 12,878 and 15,115 individual word entries, respectively (Balage Filho et al., 2013).^4^ In particular, we focused on examining the relationship between the emotional valence (LIWC’s calculated percentage of positive and negative words present) of the response with student’s psychological experiences in graduate school (i.e., social belonging, threat, and challenge). In addition, we also examined how support from graduate programs during the COVID-19 pandemic were associated with psychological experiences. To address this more specific question, we utilized qualitative content analysis to thematically hand-code students’ open-ended responses (Schreier, 2014). After reading all participant responses, the third author developed a coding scheme indicating the presence (1) or absence (0) of COVID-19 impacts in multiple domains of participant’s lives (e.g., graduate work, family, mental health, physical health). This coding scheme was corroborated by two independent research assistants who developed their own coding schemes for comparison; the final coding scheme reflects the iterative discussion and agreement of coding categories by the research team (see Supplementary Material for full coding categories and results).

## Results

### Correlations

Positive psychological experiences in graduate school, defined as increased social belonging, increased challenge (motivation to succeed) and decreased threat (anxiety and nervousness related to one’s standing in the program) were correlated with increased well-being (life satisfaction and happiness) and better mental health (lower scores in depression and stress). Positive psychological experiences were also correlated positively with optimism during the COVID-19 pandemic. Life satisfaction and happiness were positively associated with optimism about the future during the COVID-19 pandemic while depression and stress were negatively associated. All of these relationships remained significant after controlling for political beliefs, income, and religiosity of students. These coefficients are displayed in Table 1.

**TABLE 1:** Correlation coefficients between the study’s measures.

	**1**	**2**	**3**	**4**	**5**	**6**	**7**	**8**
1. Social belonging	–	0.34	–0.25	–0.41	–0.29	0.38	0.32	0.11
2. Challenge	0.33	–	–0.51	–0.53	–0.51	0.46	0.40	0.23
3. Threat	–0.24	–0.51	–	0.60	0.60	–0.39	–0.37	–0.22
4. Depression	–0.40	–0.53	0.60	–	0.72	–0.61	–0.60	–0.30
5. Stress	–0.28	–0.51	0.60	0.72	–	–0.51	–0.52	–0.38
6. Satisfaction with life	0.37	0.45	–0.39	–0.61	–0.52	–	0.58	0.30
7. Subjective happiness	0.32	0.40	–0.37	–0.60	–0.53	0.58	–	0.31
8. Future Optimism	0.11	0.23	–0.23	–0.31	–0.39	0.30	0.32	–

*Raw bivariate correlations are depicted below the diagonal, while partial correlations controlling for political beliefs, religiosity and income are displayed above the diagonal. All coefficients are significant at *p* ≤ .001.*

### Path Model

To examine these relationships simultaneously, we computed a path model. As our exogenous or independent variables, we included the three aspects of psychological experiences in graduate school: social belonging, challenge and threat. As our intermediate variables, we included the different aspects of well-being and mental health: depression, stress, satisfaction with life, and subjective happiness. Our outcome was optimism about the future.

**FIGURE 1:**
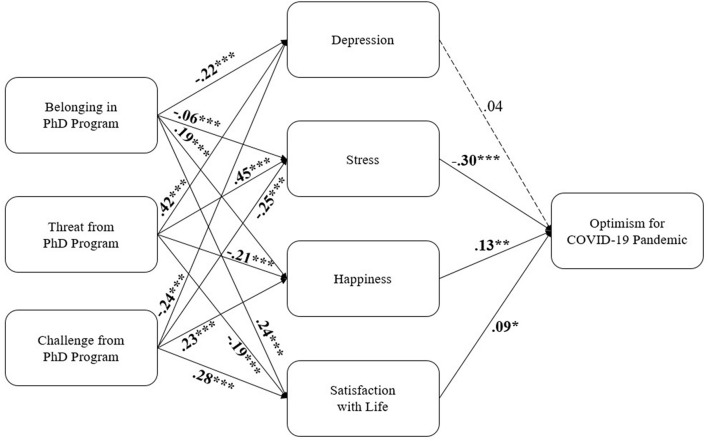
Path model for psychological experiences in graduate school predicting optimism during COVID-19 through student well-being and mental health. Standardized weights are displayed. Dashed arrows portray non-significant paths; *p ≤ .050; **p ≤ .010; ***p ≤ .001.

The model’s fit to the data was excellent: χ*^2^* (3) = 2.71, *p* = 0.438. The fit indexes also suggested a good fit: CFI = 1.00, NFI = 1.00, RMSEA < 0.001, SRMR = 0.007. All the paths were significant and in the expected direction, with the exception of the effect of depression on optimism for the future, which was no longer significant after covarying it with the other indicators of well-being and mental health. The path coefficients and the model itself are presented in Figure 1.

We also tested whether being from an underrepresented background was related to greater negative psychological experiences in graduate school, in accordance with past research findings that suggest BIPOC students feel lower social belonging, challenge, and increased threat (e.g., Steele and Aronson, 1995; Dennehy and Dasgupta, 2017). Thus, we included students’ reports of their racial, sexual and gender identities in our model as predictors of students’ psychological experiences in graduate school. We coded students’ race as ‘‘White = 1’’ and ‘‘BIPOC students = 0.’’ Though experiences vary greatly across racial and ethnic groups, given our small numbers in each racial/ethnic group, we decided to combine all BIPOC students into one group. We included sexual orientation as another variable, coded as heterosexual = 1 and LGBQA = 0. Similar to our dummy-coded race variable, we had a small number of participants who fell into each sexual minority group; thus, we combined these individuals across groups. Lastly, we coded students’ identifying gender as 1 = cisgender man and 0 = cisgender woman and transgender and nonbinary students. While transgender and nonbinary students’ experiences may vastly differ from women’s experiences, due to our small number of transgender and nonbinary students (*N* = 13), we elected to include them with cisgender women rather than removing them from the present analyses given the underrepresentation of this group in psychology.^5^ We also controlled for students’ income, religiosity, and political beliefs by future optimism on these variables.

**FIGURE 2:**
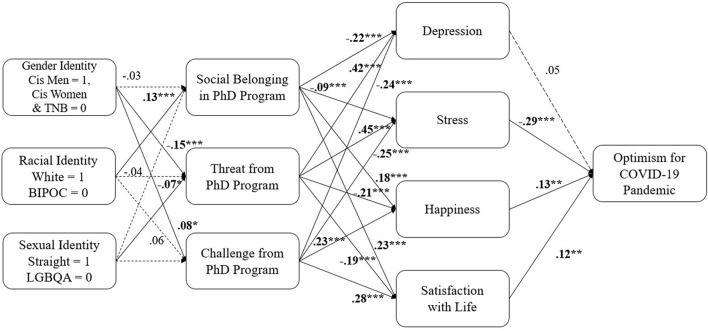
Path model showing the effect of students’ identities on their psychological experiences in graduate school predicting optimism during COVID-19 through student well-being and mental health. Cis, Cisgender; BIPOC, Black, Indigenous, and People of Color; LGBQA, Lesbian, Gay, Bisexual, Queer, and Asexual; TNB, transgender and nonbinary. Political beliefs, religiosity and income are also included as predictors of future optimism, to account for their effect on our outcome; Standardized weights are displayed; Dashed arrows portray non-significant paths; values are significant at *p ≤ .050; **p ≤ .010; ***p ≤ .001.

This model also exhibited very good fit to the data, albeit worse than Figure 1: χ*^2^* (39) = 98.90, *p* < 0.001; CFI = 0.98, NFI = 0.97, RMSEA = 0.04, SRMR = 0.03. All the paths from Figure 1 remained significant. From the demographic variables, sexual orientation was associated with greater threat (β = −0.07, SE = 0.03, *p* < 0.05), such that those who identified as heterosexual reported less threat than LGBQA students. Race was correlated with social belonging (β = 0.13, SE = 0.03, *p* < 0.001), such that White students reported greater belonging than students of color. Gender was associated with both threat (β = −0.15, SE = 0.03, *p* < 0.001) and challenge (β = 0.08, SE = 0.03, *p* < 0.05), such that women, transgender, and nonbinary students expressed greater threat and lower challenge in their graduate programs. All the path coefficients for this model are displayed in Figure 2.

### Qualitative Evidence

As expected, LIWC analysis indicated that greater social belonging and challenge were each correlated with a lower percentage of negative emotion words within a participant’s open-ended response to the impact of COVID-19 on their lives. In contrast, greater threat was correlated with a higher percentage of negative emotion words in addition to a lower percentage of positive emotion words. Furthermore, depression and stress were positively correlated with the percentage of negative emotion words, while happiness was negatively correlated with the percentage of negative emotion words. Greater optimism was correlated with a lower percentage of negative emotion words; it was also correlated with a higher percentage of positive emotion words (see Table 2).

**TABLE 2:** Correlation coefficients with LIWC variables.

	**Positive emotions**	**Negative emotions**
Social belonging	0.03	−−0.08*
Challenge	0.03	−−0.11***
Threat	−−0.07*	0.09**
Depression	–0.03	0.09*
Stress	–0.04	0.09**
Satisfaction with life	0.05	–0.05
Subjective happiness	0.00	−−0.09**
Future optimism	0.09*	−−0.12***

Values are significant at *p ≤ .050; **p ≤ .010; ***p ≤ .001. Bolded values highlight significant associations.

In addition, qualitative content coding indicated that students who specifically described increased pressure and work from their programs due to COVID-19 reported lower levels of challenge and happiness as well as higher levels of threat and stress. In contrast, students who described increased program support from their programs because of COVID-19 reported lower levels of stress and greater feelings of belonging, challenge, life satisfaction, and, most importantly, optimism (see Table 3). Examples of a supportive and an unsupportive response from one’s graduate program are presented below:

**TABLE 3:** Correlation coefficients with qualitative content codes of increased program pressure and increased program support during COVID-19.

	**Increased program**	**Increased program**
	**pressure due to**	**support due to**
	**COVID-19**	**due to COVID-19**
Social belonging	–0.05	0.11*
Challenge	−−0.11*	0.13**
Threat	0.11**	–0.05
Depression	0.07	–0.08
Stress	0.12**	−−0.11*
Satisfaction with life	–0.03	0.13**
Subjective happiness	−−0.11*	0.08
Future optimism	–0.04	0.10*

Values are significant at *p ≤ .050; **p ≤ .010. Bolded values highlight significant associations.

Supportive program response:

*I value face-to-face interaction which is no longer happening, and executing my teaching assistant duties now looks very different than it did a few weeks ago. But, overall, people have been supportive and communicative, and my program has ultimately kept me sane throughout this ordeal.* – Student 185

Unsupportive program response:

*The constant pressure to produce work and ignore our mental well-being is more pervasive now than ever before. Graduate programs, as a whole, seem to be acting as though the COVID-19 pandemic is simply a required sabbatical. This is wrong and inappropriate– the COVID-19 pandemic is a pandemic. People are dying, including those we know and love. It is inappropriate, and outright negligent, to pressure students to not only proceed as though nothing has changed, but also produce their best work yet. This pressure, along with the other mental and physical tolls the pandemic has taken, are making it incredibly difficult to persevere as a student.* – Student 762

## Discussion

Graduate students experience high amounts of stress and poor mental health outcomes (Evans et al., 2018), which may be exacerbated due to the current COVID-19 pandemic (Zahneis, 2020). In a sample of 916 psychology graduate students from 144 American universities, we found that positive psychological experiences (higher belonging and challenge, and lower threat) regarding one’s graduate program were associated with better mental health outcomes (lower depression and stress), greater well-being (life satisfaction and happiness), and greater optimism about the future during the COVID-19 pandemic. Students of color, LGBTQ+ students, and women reported worse psychological experiences in their graduate programs.^6^ Furthermore, correlations between our quantitative and qualitative responses indicated a small but significant association between a student’s stress levels in graduate school during the pandemic and student’s psychological experiences and mental health outcomes. A small but significant correlation was also observed between reported increased support due to COVID-19 and more positive psychological experiences, better mental health outcomes, and greater optimism.

Our findings suggest that graduate programs could play a significant role in graduate students’ optimism during the COVID-19 pandemic. When students felt more support during the pandemic, this was associated with greater optimism. Furthermore, programs could proactively create environments that would bolster students’ optimism during periods of uncertainty. For example, when students felt less threatened by their obligations and felt that they belonged in their programs, they felt more optimistic about the pandemic. Given that we know that optimism is malleable (Peters et al., 2010), and that interventions aiming to increase optimism are often effective (Malouff and Schutte, 2016), graduate programs could strive to ensure their students maintain high levels of optimism, potentially increasing resilience during the ongoing pandemic.

Furthermore, in our sample, and that of previous studies (e.g., Steele and Aronson, 1995; Walton and Cohen, 2011; Dennehy and Dasgupta, 2017), students from underrepresented backgrounds experienced more negative psychological experiences (lower belonging, higher threat, and lower challenge), which in turn, exacerbated the negative impact of the pandemic for these students. The COVID-19 pandemic has disproportionately affected BIPOC communities in the United States, as Black, Latinx, Asian, and Native American people have a morbidity and mortality rate that is higher than White individuals’ (Center for Disease Control and Prevention, 2020; Oppel et al., 2020). Media and scholars have also acknowledged the current double pandemic, as racial discrimination has increased against Asians (Addo, 2020; Croucher et al., 2020) and Black Americans continue to face racial injustice at the hands of law enforcement (Kendi, 2020). To address these inequities, graduate programs should recognize that students of traditionally stereotyped or underrepresented backgrounds face additional challenges in graduate school which may be exacerbated by the pandemic and programs should work to rectify these disparities (e.g., increase diversity in their programs, provide more resources and funding for BIPOC students).

## Limitations

We also note a few limitations in our study. First and foremost, our results rely exclusively on correlational evidence from a single timepoint during the emergence of the COVID-19 pandemic in the United States. Although informative, these findings cannot shed light on how graduate school psychological experiences, mental health and optimism have fluctuated during the course of the pandemic. Second, our sample did not have a large enough number of transgender and nonbinary students (*n* = 13) to analyze as a separate gender group; thus, in order to include these students in our analysis, we opted to combine their responses with cisgender females, another gender group that has been historically marginalized in the academy. However, we do note that the academic experiences of transgender and nonbinary students greatly differ from cisgender females (Goldberg et al., 2019) and future research should aim to recruit larger samples to accurately capture each group’s unique lived experiences. Finally, although our qualitative analyses provide further insights into graduate student experiences during the pandemic, our open-ended questions were rather limited in their content. Although our qualitative results are useful in validating the conceptual nature of our measurements and provide initial insights into the relationship between graduate program support in times of crisis and student well-being, these findings are limited both by the small observed effect sizes and our study’s correlational nature. Neither the LIWC analysis or hand-coding provide clarity into the direction of the relationship. In addition, while LIWC is a reputable program commonly used for text analysis, it is not possible to see what exact dictionary words are being considered in its calculated text analysis, and similarly, hand-coding qualitative data is a subjective process that may have some variation based on the coder(s). To address these concerns, future research could build on the current coding scheme by explicitly asking participants if and how COVID-19 impacted their graduate school experience, ideally examining perceptions of participants about each domain at time points both before and after COVID-19’s onset.

## Future Directions and Recommendations

Despite these limitations, we outline a few suggestions based on our results for graduate programs. First, to increase or maintain social belonging during the COVID-19 pandemic and other times of crisis in general, programs could create virtual spaces for social support and connection. For example, programs could utilize these spaces to check-in on students, to inquire about their well-being, and to provide encouragement. Faculty advisors could also check-in regularly with their graduate mentees to provide support as the pandemic progresses, as students who expressed feeling greater support from advisors felt greater belonging in their programs (Curtin et al., 2013). Second, to decrease feelings of threat and to increase feelings of challenge, programs should recognize that the current situation is unprecedented and involves heightened levels of uncontrollable stress that are not easy to overcome. Graduate students should not be expected to be more productive or to spend more time working (i.e., before the pandemic, 76% of graduate students already reported working 41 or more hours a week; Lauchlan, 2019). Since graduate students experience anxiety and depression at much higher rates than the general population (Evans et al., 2018), greater expectations in work may have detrimental impacts during the current pandemic. We recommend instead that graduate students should be allowed more flexible timelines (i.e., similar to increasing tenure clocks for faculty; Pettit, 2020) as the impact of the pandemic on student lives is prolonged.

Third, it should be recognized that each graduate student will have different circumstances as a function of their social identity (e.g., race, gender, and sexual identity) and due to individual life events. Students from underrepresented backgrounds face greater societal challenges, which can carry over into academia. The pandemic has also disproportionally affected BIPOC communities (Croucher et al., 2020; Oppel et al., 2020). Furthermore, the pandemic has negatively impacted working mothers, who are generally the primary caretakers, as they are expected to both provide childcare and continue to work (Robinson, 2020). To address these issues, graduate programs should strive to be inclusive of all of their students and do so in a way that is procedurally fair and equitable. Each student will face unique stressors, and consequently, programs and advisors should allow each student to have flexibility in their duties while also striving to maintain confidence in their students’ abilities.

In conclusion, we believe that graduate programs can help bolster graduate student mental health, well-being, and optimism during the COVID-19 pandemic through increasing social belonging and encouragement, as well as decreasing anxiety by adjusting graduate student expectations. These suggestions may be even more important for women, BIPOC, and LGBTQ+ students, as they face increased societal stressors that may also impact their experiences and productivity in graduate school.

## Data Availability Statement

The raw data supporting the conclusions of this article will be made available by the authors, without undue reservation.

## Ethics Statement

The studies involving human participants were reviewed and approved by University of Massachusetts Amherst Human Research Protection Office. The patients/participants provided their written informed consent to participate in this study.

## Author Contributions

SS: project administration, data curation, investigation, conceptualization, formal analysis, methodology, visualization, software, validation, writing—original draft, and writing—review and editing. DW: project administration, data curation, investigation, methodology, conceptualization, validation, writing—original draft, and writing—review and editing. BB: visualization, formal analysis, methodology, writing—original draft, and writing—review and editing. EM: methodology, supervision, and writing—review and editing. All authors contributed to the article and approved the submitted version.

## Conflict of Interest

The authors declare that the research was conducted in the absence of any commercial or financial relationships that could be construed as a potential conflict of interest.

## Publisher’s Note

All claims expressed in this article are solely those of the authors and do not necessarily represent those of their affiliated organizations, or those of the publisher, the editors and the reviewers. Any product that may be evaluated in this article, or claim that may be made by its manufacturer, is not guaranteed or endorsed by the publisher.

## Footnotes

1 For list of all the associations which were contacted with the purpose of posting our study’s link on their listserv, see the online Supplementary Material.

2 For a list of all the institutions for which at least one student responded to the survey see the online Supplementary Material.

3 The total number of students does not add up to 916 on specific items or demographic information, as some students left certain questions unanswered.

4 Research has demonstrated that the LIWC calculations of positive and negative emotion words correspond well to human ratings of the writing excerpts (Alpers et al., 2005) and that overall, LIWC provides accurate identification of emotion in language use (Tausczik and Pennebaker, 2010).

5 Results are identical with and without the 13 students who identified as transgender or nonbinary.

6 These analyses can be found in the online Supplementary Material.
