# Supplementary material for: Psychology Doctoral Program Experiences and Student Well-Being, Mental Health, and Optimism During the COVID-19 Pandemic
Source: Front Psychol. 2021 Sep 10;12:629205. doi: 10.3389/fpsyg.2021.629205 (PMC8460876; doi:10.3389/fpsyg.2021.629205)
Supplement: Supplementary file 1 [file Table_1.DOCX]

**Table S1**

*Number of Students Who Participated in the Survey for Each Institution*

| Name of University | Number of Students who participated |
| --- | --- |
| 1. Antioch University New England | 2 |
| 1. Ball State University | 8 |
| 1. Baylor University | 1 |
| 1. Boston University | 6 |
| 1. Bowling Green State University | 8 |
| 1. Brandeis University | 7 |
| 1. Carnegie Mellon | 1 |
| 1. Case Western Reserve University | 3 |
| 1. Chicago School of Professional Psychology | 8 |
| 1. Clark University | 3 |
| 1. Clemson University | 1 |
| 1. Columbia University | 3 |
| 1. Cornell University | 6 |
| 1. CUNY Graduate Center | 2 |
| 1. Duquesne University | 1 |
| 1. East Carolina University | 4 |
| 1. East Tennessee State University | 2 |
| 1. Emory University | 5 |
| 1. Florida Atlantic University | 11 |
| 1. Florida International University | 36 |
| 1. Florida State University | 18 |
| 1. George Mason University | 27 |
| 1. George Washington University | 6 |
| 1. Georgetown University | 3 |
| 1. Georgia Institute of Technology | 3 |
| 1. Harvard University | 1 |
| 1. Idaho State University | 5 |
| 1. Indiana University | 13 |
| 1. Indiana University (PUI) | 6 |
| 1. Iowa State University | 6 |
| 1. Jefferson University | 1 |
| 1. Kansas State University | 10 |
| 1. Lehigh University | 4 |
| 1. Michigan State University | 12 |
| 1. Midwestern University | 1 |
| 1. Montana State University | 2 |
| 1. New Mexico State University | 4 |
| 1. North Carolina State University | 18 |
| 1. North Dakota State University | 4 |
| 1. Northeastern University | 3 |
| 1. Northern Illinois University | 2 |
| 1. Northwestern University | 10 |
| 1. Oakland University | 4 |
| 1. Oklahoma State University | 4 |
| 1. Pacifica Graduate Institute | 1 |
| 1. Palo Alto University | 3 |
| 1. Pennsylvania State University | 12 |
| 1. Portland State University | 5 |
| 1. Princeton University | 4 |
| 1. Purdue University | 7 |
| 1. Rice University | 5 |
| 1. Rosalind Franklin University | 14 |
| 1. Rutgers University | 1 |
| 1. SDSU & UC San Diego Joint Program | 4 |
| 1. Southern Illinois University | 1 |
| 1. Southern Methodist University | 3 |
| 1. Stanford University | 5 |
| 1. Stony Brook University | 21 |
| 1. SUNY at Buffalo | 1 |
| 1. Texas A&M University | 7 |
| 1. Texas Christian University | 11 |
| 1. Texas Technical University | 10 |
| 1. Texas Woman's University | 2 |
| 1. Tufts University | 3 |
| 1. Tulane University | 3 |
| 1. University of Massachusetts Amherst | 6 |
| 1. University of Massachusetts Boston | 8 |
| 1. University of Massachusetts Lowell | 1 |
| 1. University at Buffalo | 8 |
| 1. University of Akron | 16 |
| 1. University of Alabama | 3 |
| 1. University of Alabama at Birmingham | 1 |
| 1. University of Alabama at Tuscaloosa | 1 |
| 1. University of Alaska Anchorage | 1 |
| 1. University of Arizona | 6 |
| 1. University of California Berkeley | 1 |
| 1. University of California Davis | 8 |
| 1. University of California Irvine | 6 |
| 1. University of California Los Angeles | 17 |
| 1. University of California Merced | 5 |
| 1. University of California San Diego | 3 |
| 1. University of California Santa Barbara | 4 |
| 1. University of California Santa Cruz | 3 |
| 1. University of Colorado Boulder | 2 |
| 1. University of Colorado Denver | 3 |
| 1. University of Connecticut | 23 |
| 1. University of Delaware | 1 |
| 1. University of Florida | 3 |
| 1. University of Georgia | 2 |
| 1. University of Houston | 4 |
| 1. University of Illinois at Chicago | 5 |
| 1. University of Illinois Urbana-Champaign | 6 |
| 1. University of Iowa | 4 |
| 1. University of Louisville | 2 |
| 1. University of Maryland College Park | 3 |
| 1. University of Miami | 1 |
| 1. University of Michigan | 19 |
| 1. University of Minnesota | 4 |
| 1. University of Mississippi | 4 |
| 1. University of Missouri | 6 |
| 1. University of Missouri at Kansas City | 1 |
| 1. University of Montana | 2 |
| 1. University of Nebraska Lincoln | 9 |
| 1. University of Nevada Las Vegas | 5 |
| 1. University of Nevada Reno | 1 |
| 1. University of Nevada Reno | 4 |
| 1. University of New Hampshire | 11 |
| 1. University of New Mexico | 5 |
| 1. University of North Carolina Chapel Hill | 12 |
| 1. University of North Carolina Charlotte | 2 |
| 1. University of North Carolina Greensboro | 12 |
| 1. University of North Dakota | 6 |
| 1. University of Notre Dame | 14 |
| 1. University of Ohio | 11 |
| 1. University of Oklahoma | 3 |
| 1. University of Oregon | 5 |
| 1. University of Pennsylvania | 1 |
| 1. University of Pittsburgh | 1 |
| 1. University of Rhode Island | 2 |
| 1. University of Rochester | 6 |
| 1. University of South Carolina | 1 |
| 1. University of South Florida | 9 |
| 1. University of Southern California | 1 |
| 1. University of Southern Mississippi | 1 |
| 1. University of Tennessee | 5 |
| 1. University of Texas at Austin | 7 |
| 1. University of Texas at El Paso | 4 |
| 1. University of Toledo | 11 |
| 1. University of Utah | 3 |
| 1. University of Virginia | 8 |
| 1. University of Washington | 3 |
| 1. University of West Georgia | 3 |
| 1. University of Wisconsin Madison | 6 |
| 1. University of Wisconsin Milwaukee | 13 |
| 1. Utah State University | 11 |
| 1. Vanderbilt University | 6 |
| 1. Virginia Tech | 7 |
| 1. Washington State University | 8 |
| 1. Washington University St. Louis | 7 |
| 1. Wayne State University | 8 |
| 1. West Virginia University | 11 |
| 1. Western Michigan University | 1 |
| 1. Wichita State University | 8 |
| 1. Yale University | 7 |
| *Did not answer/missing* | 55 |

**Table S2**

*List of Psychological Organizations Contacted*

| Organization’s name |
| --- |
| 1. [Society for General Psychology](https://www.apa.org/about/division/div1) |
| 1. [Society for the Teaching of Psychology](https://www.apa.org/about/division/div2) |
| 1. [Society for Experimental Psychology and Cognitive Science](https://www.apa.org/about/division/div3) |
| 1. Society for [Quantitative and Qualitative Methods](https://www.apa.org/about/division/div5) |
| 1. [Society for Behavioral Neuroscience and Comparative Psychology](https://www.apa.org/about/division/div6) |
| 1. Society for [Developmental Psychology](https://www.apa.org/about/division/div7) |
| 1. [Society for Personality and Social Psychology](https://www.apa.org/about/division/div8) |
| 1. [Society for the Psychological Study of Social Issues (SPSSI)](https://www.apa.org/about/division/div9) |
| 1. [Society for the Psychology of Aesthetics, Creativity and the Arts](https://www.apa.org/about/division/div10) |
| 1. [Society of Clinical Psychology](https://www.apa.org/about/division/div12) |
| 1. [Society of Consulting Psychology](https://www.apa.org/about/division/div13) |
| 1. [Society for Industrial and Organizational Psychology](https://www.apa.org/about/division/div14) |
| 1. Society for [Educational Psychology](https://www.apa.org/about/division/div15) |
| 1. Society for [School Psychology](https://www.apa.org/about/division/div16) |
| 1. [Society of Counseling Psychology](https://www.apa.org/about/division/div17) |
| 1. [Psychologists in Public Service](https://www.apa.org/about/division/div18) |
| 1. [Society for Military Psychology](https://www.apa.org/about/division/div19) |
| 1. Society for [Adult Development and Aging](https://www.apa.org/about/division/div20) |
| 1. Society for [Applied Experimental and Engineering Psychology](https://www.apa.org/about/division/div21) |
| 1. Society for [Rehabilitation Psychology](https://www.apa.org/about/division/div22) |
| 1. [Society for Consumer Psychology](https://www.apa.org/about/division/div23) |
| 1. [Society for Theoretical and Philosophical Psychology](https://www.apa.org/about/division/div24) |
| 1. Society for [Behavior Analysis](https://www.apa.org/about/division/div25) |
| 1. [Society for the History of Psychology](https://www.apa.org/about/division/div26) |
| 1. [Society for Community Research and Action: Division of Community Psychology](https://www.apa.org/about/division/div27) |
| 1. Society for [Psychopharmacology and Substance Abuse](https://www.apa.org/about/division/div28) |
| 1. [Society for the Advancement of Psychotherapy](https://www.apa.org/about/division/div29) |
| 1. [Society of Psychological Hypnosis](https://www.apa.org/about/division/div30) |
| 1. Society for [Society for Humanistic Psychology](https://www.apa.org/about/division/div32) |
| 1. Society for [Intellectual and Developmental Disabilities/Autism Spectrum Disorder](https://www.apa.org/about/division/div33) |
| 1. [Society for Environmental, Population and Conservation Psychology](https://www.apa.org/about/division/div34) |
| 1. [Society for the Psychology of Women](https://www.apa.org/about/division/div35) |
| 1. [Society for the Psychology of Religion and Spirituality](https://www.apa.org/about/division/div36) |
| 1. [Society for Child and Family Policy and Practice](https://www.apa.org/about/division/div37) |
| 1. [Society for Health Psychology](https://www.apa.org/about/division/div38) |
| 1. [Society for Psychoanalysis and Psychoanalytic Psychology](https://www.apa.org/about/division/div39) |
| 1. [Society for Clinical Neuropsychology](https://www.apa.org/about/division/div40) |
| 1. [American Psychology-Law Society](https://www.apa.org/about/division/div41) |
| 1. [Psychologists in Independent Practice](https://www.apa.org/about/division/div42) |
| 1. [Society for Couple and Family Psychology](https://www.apa.org/about/division/div43) |
| 1. [Society for the Psychology of Sexual Orientation and Gender Diversity](https://www.apa.org/about/division/div44) |
| 1. [Society for the Psychological Study of Culture, Ethnicity and Race](https://www.apa.org/about/division/div45) |
| 1. [Society for Media Psychology and Technology](https://www.apa.org/about/division/div46) |
| 1. [Society for Sport, Exercise and Performance Psychology](https://www.apa.org/about/division/div47) |
| 1. [Society for the Study of Peace, Conflict and Violence: Peace Psychology Division](https://www.apa.org/about/division/div48) |
| 1. [Society of Group Psychology and Group Psychotherapy](https://www.apa.org/about/division/div49) |
| 1. [Society of Addiction Psychology](https://www.apa.org/about/division/div50) |
| 1. [Society for the Psychological Study of Men and Masculinities](https://www.apa.org/about/division/div51) |
| 1. Society for [International Psychology](https://www.apa.org/about/division/div52) |
| 1. [Society of Clinical Child and Adolescent Psychology](https://www.apa.org/about/division/div53) |
| 1. [Society of Pediatric Psychology](https://www.apa.org/about/division/div54) |
| 1. [American Society for the Advancement of Pharmacotherapy](https://www.apa.org/about/division/div55) |
| 1. Society for [Trauma Psychology](https://www.apa.org/about/division/div56) |
| 1. Association for Psychological Science |
| 1. Society for Research in Child Development |
| 1. Psi Chi Eastern Region |
| 1. Psi Chi Midwestern Region |
| 1. Psi Chi Rocky Mountain Region |
| 1. Psi Chi Southeastern Region |
| 1. Psi Chi Southwestern Region |
| 1. Psi Chi Western Region |

**Detailed Demographic Information**

- Born in USA
  - Yes = 772 (85%)
  - No = 137 (15%)
- Age
  - *M_age_* = 27.73, *SD_age_* = 4.16
- Is a parent
  - Yes = 54 (6%)
  - No = 854 (94%)
- Year in Graduate school (199 missing)
  - 1 year = 155 (22%)
  - 2 years = 189 (26%)
  - 3 years = 14 (2%)
  - 4 years = 164 (23%)
  - 5 years = 131 (18%)
  - More than 5 years = 64 (9%)
- Gender (Missing = 9)
  - Man = 193 (21%)
  - Woman = 701 (77%)
  - Other = 13 (1%)
- Race (17 missing)
  - White American = 645 (72%)
  - African American or Black = 37 (4%)
  - LatinX = 59 (6%)
  - East Asian/Asian American = 107 (12%)
  - Multiracial = 48 (5%)
  - Other = 3 (< 1%)
- Sexual Orientation (Missing = 28)
  - Straight = 650 (73%)
  - Gay/lesbian = 45 (5%)
  - Bisexual = 188 (21%)
  - Asexual and Other = 5 (< 1%)
- Program of Study
  - Clinical = 321 (35%)
  - Cognitive = 96 (11%)
  - Counseling = 32 (4%)
  - Developmental = 86 (9%)
  - Industrial and Organizational = 52 (6%)
  - Neuroscience = 67 (7%)
  - Social and personality = 127 (14%)
  - Other = 127 (14%)

**Social Identity Comparisons**

We performed all analyses in SAS (Statistical Analysis System). Specifically, for the demographic comparisons we used the general linear model (GLM) procedure for t-tests, analyses of variance as well as moderated regressions. This procedure outputs F instead of t values. Any corresponding t values can be determined according to *F* = *t^2^*. For the omnibus effects see Table S3. For the means and SDs for each comparison see Tables

S4-S6.

**Table S3.**

*Omnibus Effects for the Social Identity Comparisons*

| Measure | Gender  Identity | Racial  Identity | Sexual  Orientation |
| --- | --- | --- | --- |
| Depression | F (1, 903) = 2.40, *p* = .121, η^2^ = .003 | **F (1, 90) = 8.86, *p* = .003, η^2^ = .010** | **F (1, 906) = 12.35, *p* < .001, η^2^ = .013** |
| Stress | **F (1, 903) = 23.08, *p* < .001, η^2^ = .025** | F (1, 906) = 2.05, *p* = .152, η^2^ = .002 | **F (1, 906) = 11.89, *p* < .001, η^2^ = .013** |
| Satisfaction with Life | F (1, 903) = 0.20, *p* = .655, η^2^ < .001 | **F (1, 906) = 12.16, *p* < .001, η^2^ = .013** | **F (1, 906) = 5.61, *p* = .018, η^2^ = .006** |
| Happiness | F (1, 902) = 0.01, *p* = .903, η^2^ < .001 | F (1, 905) = 2.63, *p* = .105, η^2^ = .003 | **F (1, 905) = 9.22, *p* = .002, η^2^ = .010** |
| Belonging | F (1, 901) = 1.28, *p* = .258, η^2^ = .001 | **F (1, 904) = 18.34, *p* < .001, η^2^ = .020** | F (1, 904) = 2.92, *p* = .088, η^2^ = .003 |
| Threat | **F (1, 903) = 11.67, *p* < .001, η^2^ = .020** | F (1, 906) = 1.67, *p* = .197, η^2^ = .002 | **F (1, 906) = 5.40, *p* = .020, η^2^ = .006** |
| Challenge | **F (1, 902) = 7.04, *p* = .008, η^2^ = .008** | F (1, 905) = 2.50, *p* = .114, η^2^ = .003 | **F (1, 905) = 4.62, *p* = .032, η^2^ = .005** |
| Optimism for COVID-19 | F (1, 883) = 1.5, *p* = .211, η^2^ = .002 | F (1, 885) = 0.07, *p* = .787, η^2^ < .001 | **F (1, 885) = 11.77, *p* < .001, η^2^ = .013** |

*Note:* Bolded values depict significant results.

**Tables S4-S6**

*Social Identity Comparisons for the Three Different Identities*

| Table S4:  Gender identity | Depression | | Stress | | Happiness | | Satisfaction with Life | | Belonging | | Threat | | Challenge | | Optimism | |
| --- | --- | --- | --- | --- | --- | --- | --- | --- | --- | --- | --- | --- | --- | --- | --- | --- |
|  | *M* | *SD* | *M* | *SD* | *M* | *SD* | *M* | *SD* | *M* | *SD* | *M* | *SD* | *M* | *SD* | *M* | *SD* |
| Woman & TNB  (*N* = 711) | 4.85 | 1.45 | **5.32** | **1.30** | 5.66 | 1.73 | 5.71 | 1.63 | 6.34 | 2.00 | **6.33** | **1.43** | **6.21** | **1.55** | 4.27 | 1.97 |
| Man (*N* = 192) | 4.67 | 1.53 | **4.80^b^** | **1.52** | 5.68 | 1.85 | 5.65 | 1.72 | 6.16 | 1.91 | **5.81^b^** | **1.66** | **6.54^a^** | **1.42** | 4.29 | 2.19 |

*Note:* TNB = Transgender and Nonbinary
Bolded values depict significant differences

| Table S5: Racial identity | Depression | | Stress | | Happiness | | Satisfaction with Life | | Belonging | | Threat | | Challenge | | Optimism | |
| --- | --- | --- | --- | --- | --- | --- | --- | --- | --- | --- | --- | --- | --- | --- | --- | --- |
|  | *M* | *SD* | *M* | *SD* | *M* | *SD* | *M* | *SD* | *M* | *SD* | *M* | *SD* | *M* | *SD* | *M* | *SD* |
| BIPOC  (*N* = 192) | **5.05** | **1.55** | 5.32 | 1.47 | 5.51 | 1.84 | **5.39** | **1.72** | **5.86** | **2.09** | 6.32 | 1.56 | 6.15 | 1.59 | 4.30 | 2.10 |
| White  (*N* = 700) | **4.73** | **1.43** | 5.18 | 1.32 | 5.72 | 1.73 | **5.81** | **1.62** | **6.48** | **1.91** | 6.18 | 1.47 | 6.33 | 1.50 | 4.34 | 2.00 |

*Note:* BIPOC = Black Indigenous and People of Color.
Bolded values depict significant differences

| Table S6: Sexual orientation | Depression | | Stress | | Happiness | | Satisfaction with Life | | Belonging | | Threat | | Challenge | | Optimism | |
| --- | --- | --- | --- | --- | --- | --- | --- | --- | --- | --- | --- | --- | --- | --- | --- | --- |
|  | M | SD | M | SD | M | SD | M | SD | M | SD | M | SD | M | SD | M | SD |
| LGBQA  (*N* = 259) | **5.09** | **1.43** | **5.46** | **1.28** | **5.38** | **1.72** | **5.48** | **1.72** | 6.12 | 2.00 | **6.40** | **1.39** | **6.11** | **1.55** | **3.96** | **2.01** |
| Straight  (*N* = 647) | **4.71** | **1.48** | **5.12** | **1.39** | **5.77** | **1.77** | **5.77** | **1.62** | 6.37 | 1.97 | **6.15** | **1.53** | **6.35** | **1.52** | **4.47** | **2.01** |

*Note:* LGBQA = Lesbian, Gay, Bisexual, Queer, Asexual.
Bolded values depict significant differences.

**Table S7**

*Standardized Indirect Effects from Figures 1-2*

|  | Figure 1 | | |  | Figure 2 | | |
| --- | --- | --- | --- | --- | --- | --- | --- |
|  | *β* | *SE* | *p* |  | *β* | *SE* | *p* |
| Belonging 🡪 Well-Being 🡪 Optimism | 0.07 | 0.01 | <.001 |  | 0.06 | 0.01 | <.001 |
| Challenge 🡪 Well-Being 🡪Optimism | 0.12 | 0.01 | <.001 |  | 0.12 | 0.01 | <.001 |
| Threat 🡪 Well-Being 🡪Optimism | -0.16 | 0.02 | <.001 |  | -0.16 | 0.02 | <.001 |
| Gender Identity 🡪 Psychological Experiences 🡪Depression |  |  |  |  | -0.07 | 0.02 | .001 |
| Gender Identity 🡪 Psychological Experiences 🡪 Happiness |  |  |  |  | 0.04 | 0.02 | .009 |
| Gender Identity 🡪Psychological Experiences 🡪 Stress |  |  |  |  | -0.08 | 0.02 | <.001 |
| Gender Identity 🡪 Psychological Experiences 🡪 Life Satisfaction |  |  |  |  | 0.04 | 0.02 | .020 |
| Gender Identity 🡪 Psychological Experiences 🡪Well-Being 🡪 Optimism |  |  |  |  | 0.03 | 0.01 | .001 |
| Racial Identity 🡪 Psychological Experiences 🡪 Depression |  |  |  |  | -0.06 | 0.02 | .008 |
| Racial Identity 🡪 Psychological Experiences 🡪 Happiness |  |  |  |  | 0.05 | 0.02 | .004 |
| Racial Identity 🡪 Psychological Experiences 🡪 Stress |  |  |  |  | -0.05 | 0.02 | .038 |
| Racial Identity 🡪 Psychological Experiences 🡪 Life Satisfaction |  |  |  |  | 0.05 | 0.02 | .002 |
| Racial Identity 🡪 Psychological Experiences 🡪 Well-Being 🡪 Optimism |  |  |  |  | 0.02 | 0.01 | .017 |
| Sexual Orientation 🡪 Psychological Experiences 🡪 Depression |  |  |  |  | -0.05 | 0.02 | .017 |
| Sexual Orientation 🡪 Psychological Experiences 🡪 Happiness |  |  |  |  | 0.04 | 0.02 | .021 |
| Sexual Orientation 🡪 Psychological Experiences 🡪 Stress |  |  |  |  | -0.05 | 0.02 | .019 |
| Sexual Orientation 🡪 Psychological Experiences 🡪 Life Satisfaction |  |  |  |  | 0.04 | 0.02 | .023 |
| Sexual Orientation 🡪 Psychological Experiences 🡪 Well-Being 🡪 Optimism |  |  |  |  | 0.02 | 0.01 | .020 |

*Note.* Well-Being comprises Depression, Happiness, Stress and Life Satisfaction, and Psychological Experiences comprise Belonging, Challenge and Threat.

**Qualitative Coding of Open-Ended Responses**

Student open-ended responses were hand coded for the presence (1) or absence (0) of five different areas in which the COVID-19 pandemic may have impacted their life. These were: Financial/Career, Graduate Work (beyond the impact of simply working from a remote location), Others’ Well-being, Family, Mental Health, and Physical Health. These results are displayed in Figure S1.

**Figure S1**

*Percentages of Responses Given by Graduate Students for the Impact of COVID-19 on Their Lives*

Due to the high prevalence of responses that indicated impacts to graduate work (56.77% percent of total student responses), a more in-depth coding was implemented to analyze the presence (1) or absence (0) of different domains of impact relevant to the graduate experience. These were: Disrupted Research Progress, Disrupted Degree Progress, Disrupted Internship/Practicum, Online Challenges for Teaching/Learning/Clinical Practice, Decreased Motivation/Productivity, Decreased Peer Support, Increased Program Support, Increased Program Pressure, Conference Cancellation, and New Study Adaptation.

**Figure S2**

*Breakdown of Percentages for the Responses of the Impact of COVID-19 on Graduate Students’ Work*
